# KIAA1199 is induced by inflammation and enhances malignant phenotype in pancreatic cancer

**DOI:** 10.18632/oncotarget.15052

**Published:** 2017-02-04

**Authors:** Shiro Kohi, Norihiro Sato, Atsuhiro Koga, Nobutaka Matayoshi, Keiji Hirata

**Affiliations:** ^1^ Department of Surgery 1, University of Occupational and Environmental Health, Kitakyushu, Fukuoka 807-8555, Japan

**Keywords:** pancreatic cancer, KIAA1199, hyaluronan, migration, inflammation

## Abstract

**Background:**

Recent evidence suggests a critical role of hyaluronan (HA), especially low-molecular-weight HA (LMW-HA), in the aggressive tumor phenotype. Increased expression of KIAA1199, a newly identified protein involved in HA degradation, has been reported in various cancers, including pancreatic ductal adenocarcinoma (PDAC). However, little is known about the functional significance of KIAA1199 in PDAC.

**Methods:**

Using siRNA knockdown and forced expression models, we investigated the effects of KIAA1199 expression on malignant behaviors (proliferation, migration, and invasion) of PDAC cells. We also examined the effect of inflammation on the transcriptional regulation of KIAA1199 using a pro-inflammatory cytokine and anti-inflammatory agent.

**Results:**

Knockdown of KIAA1199 expression using siRNA resulted in decreased cell migration and proliferation. On the other hand, forced expression of KIAA1199 using gene transduction significantly enhanced the migration and invasion. Importantly, increased KIAA1199 expression was associated with an increased level of LMW-HA in the conditioned medium. Exposure to a pro-inflammatory cytokine, interleukin-1ß, increased the KIAA1199 transcription and enhanced the migration. In contrast, treatment with NS-398, a cyclooxygenase-2 inhibitor, decreased the KIAA1199 expression and inhibited the migration.

**Conclusions:**

These findings suggest that increased KIAA1199 expression may contribute to the aggressive phenotype partly through increasing the LMW-HA concentration. Our present results also suggest a possible link between inflammation, induced KIAA1199 expression, and enhanced migration during PDAC progression.

## INTRODUCTION

Pancreatic ductal adenocarcinoma (PDAC) is one of the most aggressive neoplasms ranking the fourth among cancer-related deaths in western countries. The prognosis of patients with PDAC remains dismal with the overall 5-year survival rate of less than 5% [1]. One major reason responsible for this extremely poor prognosis in PDAC is related to its rapid invasion to the surrounding structures and metastatic spread to the lymph nodes and distant organs. Therefore, understanding the biological and molecular mechanisms underlying this aggressive tumor phenotype is important to develop novel therapeutic strategies and improve survival of patients with PDAC.

Recently, the study focus in the field of PDAC research has shifted from cancer cells themselves to their surrounding microenvironment. PDAC is characterized by a dense stroma consisting of various stromal cells (including inflammatory cells, stellate cells, and activated fibroblasts) embedded within rich extracellular matrix (ECM) [2]. Hyaluronan (HA), a major component of ECM, is abundantly accumulated in a wide range of cancers including PDAC and plays a critical role in a variety of cellular processes including cell proliferation, migration, invasion, metastasis, angiogenesis, and resistance to chemotherapeutic agents [3–8]. Interestingly, low-molecular-weight HA (LMW-HA) or small HA fragments, rather than high-molecular-weight HA (HMW-HA), has been suggested to be essential for cancer progression in terms of invasion and metastasis [9, 10]. We also show that LMW-HA stimulates motility of PDAC cells [11]. HA, a large linear glycosaminoglycan of up to 10^6^-10^7^ Da in its naïve form, is produced by HA-synthesizing enzymes (HASs) and degraded into smaller fragments by hyaluronidases (HYALs). We previously demonstrated overexpression of HAS2 (one of the major HA-synthesizing proteins) in PDAC [12]. In addition to the HA synthesis, degradation of HA by HYAL is also accelerated in malignant tumors. In our previous study, HYAL1 has been shown to be overexpressed in PDAC [13]. Because of the important role of LMW-HA in the malignant phenotype, other mechanisms for HA degradation are likely to exist independent of HYALs.

*KIAA1199* is a gene originally identified in relation with non-syndromic hearing loss [14]. Previous studies suggests that KIAA1199 (also termed as CEMIP) is overexpressed, prognostic, and correlated with the malignant behavior in a variety of cancers [15–21]. Recently, KIAA1199 was found to have an ability to degrade HA independent of HYAL activities [22], suggesting a possible linkage between KIAA1199 expression, HA degradation, and cancer progression.

The aim of the present study was to investigate the functional significance of KIAA1199 in PDAC. We also examined the effect of inflammation on the KIAA1199 expression and migration of PDAC cells. Our present results suggest that KIAA1199 plays an important role in the aggressive tumor phenotype in PDAC partly through accelerating the process of HA degradation.

## RESULTS

### Knockdown of KIAA1199 expression decreased migration and proliferation in PDAC cells

**Figure 1:**
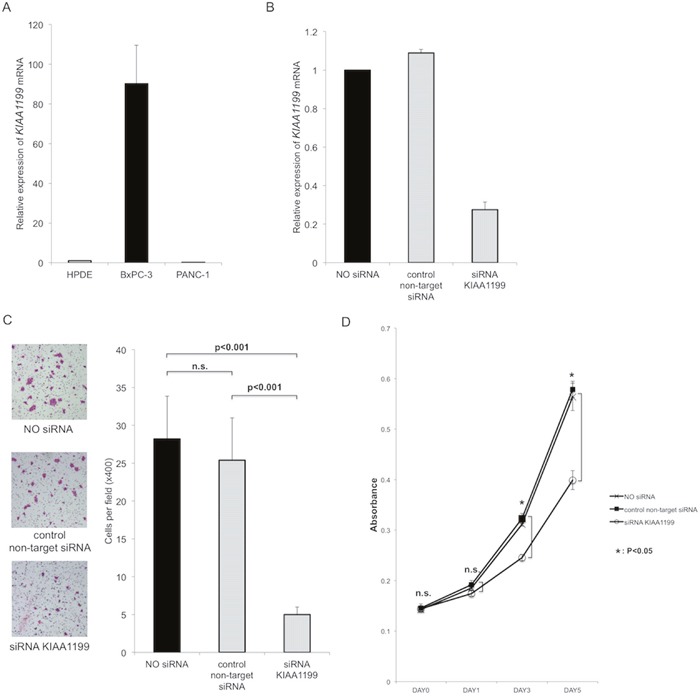
Knockdown of KIAA1199 expression decreased migration and proliferation in PDAC cells **A**. Expression of *KIAA1199* mRNA expression in PDAC cells was examined by real-time RT-PCR. **B**. Real-time RT-PCR showed that transfection with siRNA targeting for *KIAA1199* (siRNA-KIAA1199) resulted in 73% knockdown. **C**. The transwell migration assay showed that knockdown of KIAA1199 significantly inhibited the migration of PDAC cells as compared to control (non-target siRNA transfection) (P<0.001). **D**. The MTT assay showed the knockdown of KIAA1199 significantly decreased the proliferation of PDAC cells as compared to control on day3 and day5 (P<0.05).

In the present study, we used two PDAC cell lines with high and low *KIAA1199* mRNA expression (as compared to a control cell line HPDE) (Figure 1A). First, we used siRNA to knockdown the expression of KIAA1199 in BxPC-3 with high *KIAA1199* mRNA expression (Figure 1A). Real-time RT-PCR showed that transfection with siRNA targeting for *KIAA1199* (siRNA-KIAA1199) resulted in 73% knockdown (Figure 1B).

First, we investigated whether KIAA1199 affected cell migration. The transwell migration assay showed that knockdown of KIAA1199 significantly inhibited the migration of PDAC cells as compared to control (non-target siRNA transfection) (P<0.001; Figure 1C). Next, we examined whether KIAA1199 knockdown affected PDAC cell proliferation. The MTT assay showed the knockdown of KIAA1199 significantly decreased the proliferation of PDAC cells as compared to control on day3 and day 5 (P<0.05; Figure 1D).

### Upregulation of KIAA1199 expression increased migration, invasion and LMW-HA in PDAC cells

**Figure 2:**
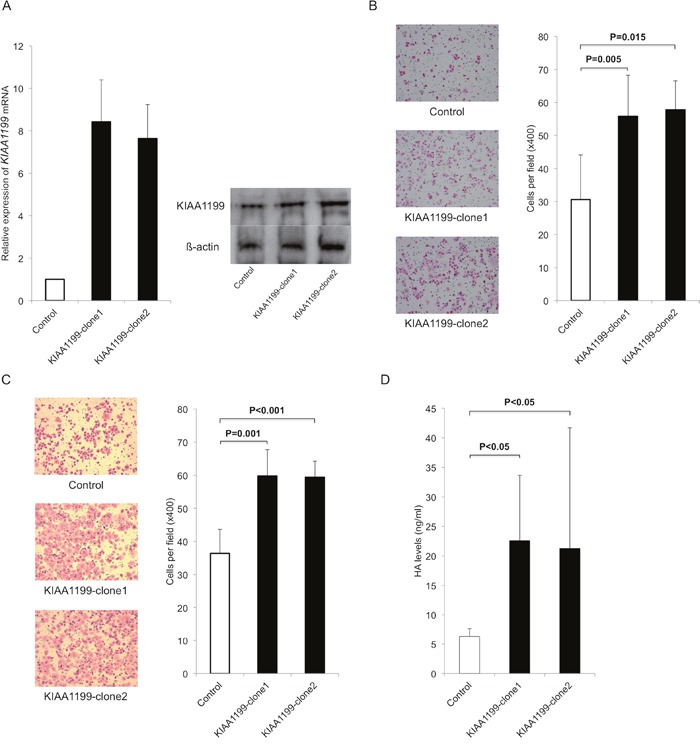
Upregulation of KIAA1199 expression increased migration, invasion and LMW-HA in PDAC cells **A**. We transfected KIAA1199 expression vector (KIAA1199-clone1, KIAA1199-clone2) or an empty vector control into PANC-1 and stable transfectants were established under selection with G418. Real-time RT-PCR and Western blot analysis validated the successful transfection and the expression of KIAA1199 mRNA and protein were upregulated in KIAA1199-clone1 and KIAA1199-clone2. **B**. The transwell migration assay showed that forced expression of KIAA1199 significantly stimulated the migration of PDAC cells as compared to control (P=0.005 for KIAA1199-clone1, P=0.015 for KIAA1199-clone2). **C**. Invasion assay showed forced expression of KIAA1199 significantly stimulated the invasion of PDAC cells as compared to control (P=0.001 for KIAA1199-clone1, P<0.001 for KIAA1199-clone2). **D**. We observed upregulation of KIAA1199 expression significantly increased the LMW-HA concentration as compared to control (P<0.05).

We next used an expression vector to examine the forced expression of KIAA1199 in PANC-1 with low expression level (Figure 1A). We transfected KIAA1199 expression vector (KIAA1199-clone1, KIAA1199-clone2) or an empty vector control into PANC-1 and stable transfectants were established under selection with G418. The KIAA1199 mRNA and protein expression were examined by real-time RT-PCR and western blot analysis, respectively. As expected, the *KIAA1199* mRNA expression was higher in the KIAA1199-clone1 and KIAA1199-clone2 (with a maximum of approximately 11-fold) as compared to the empty vector control (Figure 2A). KIAA1199 protein expression was also higher in the KIAA1199-clone1 and KIAA1199-clone2 as compared to control (Figure 2A).

We then compared abilities for migration and invasion between KIAA1199-clones and control. The transwell migration assay showed that forced expression of KIAA1199 significantly enhanced the migration of PDAC cells (P=0.005 for KIAA1199-clone1, P=0.015 for KIAA1199-clone2 as compared to control; Figure 2B). Invasion assay showed that forced expression of KIAA1199 significantly enhanced the invasion of PDAC cells (P=0.001 for KIAA1199-clone1, P<0.001 for KIAA1199-clone2 as compared to control; Figure 2C). We also examined whether overexpression of KIAA1199 affected PDAC cell proliferation. However, there was no significant difference in cell proliferation between KIAA1199 clones and control cells (date not shown).

Because KIAA1199 is involved in HA degradation, we measured the concentration of LMW-HA (<100 kDa) in the conditioned media in these cells. The concentrations of LMW-HA were significantly higher in KIAA1199-clones relative to the control (P<0.05; Figure 2D).

### Effects of inflammation on KIAA1199 expression and migration

**Figure 3:**
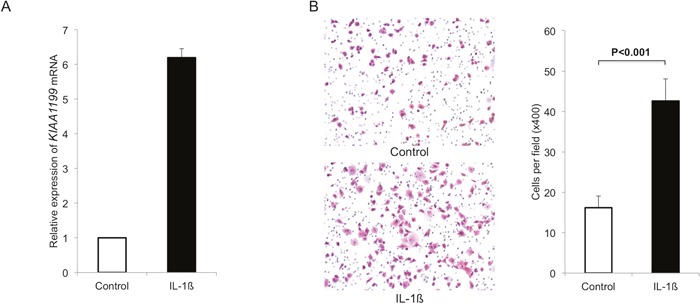
IL-1ß induces KIAA1199 expression and migration in PDAC cells **A**. We used a pro-inflammatory cytokine IL-1ß to induce inflammation. Exposure of PANC-1 cells with IL-1ß at 500 pg/ml for 48 hours robustly increased the expression of KIAA1199 (6.3-fold increase). **B**. Exposure to IL-1ß (500 pg/ml) significantly increased the number of migrating cells (P<0.001).

Because inflammation is a key factor promoting cancer initiation and progression, we thought to determine the effects of inflammation on the KIAA1199 expression in PDAC cells. First, we used a pro-inflammatory cytokine interleukin-1ß (IL-1ß) to induce inflammation. Exposure of PANC-1 cells with IL-1ß at 500 pg/ml for 48 hours robustly increased the expression of KIAA1199 (6.3-fold increase, Figure 3A).

We also examined the effect of IL-1ß on migration in PANC-1. Exposure to IL-1ß significantly increased the number of migrating cells (P<0.001; Figure 3B).

**Figure 4:**
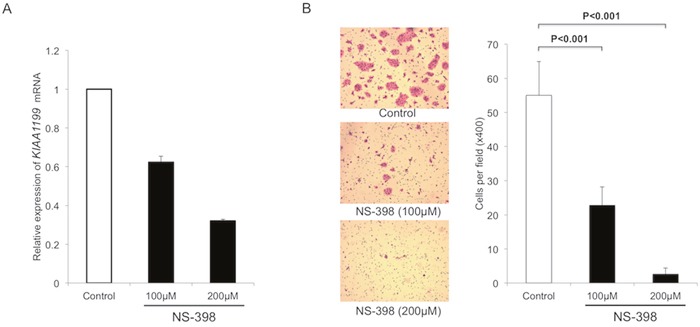
NS-398 decrease KIAA1199 expression and migration in PDAC cells **A**. Treatment with NS-398 decreased the expression of *KIAA1199* mRNA in a dose-dependent manner. **B**. After 24 h incubation, the transwell migration assay showed that NS-398 significantly inhibited the migration of BxPC-3 in a dose-dependent manner (P<0.001).

We used an anti-inflammatory drug (cyclooxygenase-2 (COX-2) inhibitor, NS-398) to further examine the effect of inflammation on the KIAA1199 expression in another PDAC cell line BxPC-3. Treatment with NS-398 decreased the expression of *KIAA1199* mRNA in a dose-dependent manner (Figure 4A). We also examined the effect of NS-398 on migration in BxPC-3. The transwell migration assay at 24 hours after treatment (when there was no significant difference in the number of cells) showed that NS-398 inhibited the migration of BxPC-3 in a dose-dependent manner (Figure 4B).

## DISCUSSION

In the present study, we investigated the effects of KIAA1199 expression on the malignant phenotype of PDAC cells by modulating its expression level. The major findings obtained were as follows: (1) knockdown of KIAA1199 resulted in decreased proliferation and migration in BxPC-3; (2) forced expression of KIAA1199 resulted in increased migration and invasion in PANC-1; (3) increased expression of KIAA1199 was associated with an elevated level of LMW-HA; and (4) KIAA1199 expression and migratory ability were increased in response to a pro-inflammatory stimulus but decreased by treatment with an anti-inflammatory drug. These findings suggest that KIAA1199, which can be induced by inflammation, may contribute to the malignant phenotype in PDAC cells partly through increasing the LMW-HA level.

The exact mechanisms by which KIAA1199 accelerates cancer progression remain undermined. However, functional studies have revealed a number of cancer-promoting effects of KIAA1199. For example, KIAA1199 is known to increase the migration of cancer cells partly through mediating endoplasmic reticulum calcium leakage, which results in protein kinase C**α** activation [23]. Previous studies have shown that KIAA1199 promotes EMT, which is associated with a more aggressive phenotype in various cancers [23]. KIAA1199 promotes cancer cell survival through EGFR signaling [24] and through promoting glycogen breakdown and preventing apoptosis [25]. The shRNA-mediated knockdown of KIAA1199 in breast cancer cells resulted in reduced cell motility and proliferation *in vitro* and decreased tumor growth in mice [15]. Genetically-engineered knockout of KIAA1199 reduces the ability of colon cancer cells to form xenograft tumors in athymic mice [19]. In the present study, we demonstrate that KIAA1199 enhances the malignant behaviors of PDAC cells in associated with an increased level of LMW-HA. Although further studies are required, our results suggest that KIAA1199 may contribute to the aggressive tumor phenotype through accelerating the HA degradation and producing a larger amount of LMW-HA.

Factors that regulate KIAA1199 expression remain poorly understood. Previous studies demonstrate that hypoxia promotes colon cancer dissemination through up-regulation of KIAA1199 [26]. In the present study, we demonstrate, for the first time, that exposure to IL-1ß induces the expression of KIAA1199 which is associated with enhanced migration. We also found that treatment with NS-398 resulted in decreased expression of KIAA1199. These findings suggest a possible mechanistic linkage between inflammation and KIAA1199 expression. In support of this finding, a previous study showed that NF-κB induces the expression of KIAA1199 and promote survival through EGFR signaling [24]. Taken together, these findings led to a hypothesis that inflammation promotes cancer initiation and progression partly through induction of KIAA1199.

In conclusion, these findings suggest that KIAA1199 expression may contribute to the aggressive phenotype of PDAC cells through increasing LMW-HA. Our present results also highlight the importance of KIAA1199 as a therapeutic target for PDAC.

## MATERIALS AND METHODS

### Cell culture and reagents

We used 2 PDAC cell lines, BxPC-3 and PANC-1 (American Type Culture Collection, Manassas, VA, USA). An immortalized cell line derived from human pancreatic duct, HPDE, was a kind gift from Dr. M.S. Tsao (Univ. of Toronto, Canada). PDAC cell lines were maintained in RPMI1640 medium (Life Technologies, Grand Island, NY, USA) supplemented with 10% fetal bovine serum (FBS) (Life Technologies) and 1% streptomycin and penicillin (Life Technologies). HPDE was maintained in HuMedia-KG2 (KURABO, Osaka, Japan), in a 5% CO_2_ incubator at 37°C. Recombinant human IL-1ß (PEPROTECH, Rocky Hill, NJ, USA) and NS-398 (Sigma-Aldrich, St.Louis, MO, USA) were used at different concentrations (IL-1ß: 500pg/ml, NS-398: 100, 200μM) for 48 hours treatment.

### siRNA targeting for KIAA1199

The siRNA targeting for KIAA1199 (ON-TARGETplus Smart Pool Human KIAA1199 L-022291-00) and negative control siRNA (ON-TARGETplus Control siRNA Non-Targeting siRNA #1 D-001810-01-05) were purchased from GE healthcare (Buckinghamshire, England). BxPC-3 was transfected with 100 nM siRNA using DhermaFECT 1 Transfection Reagent (GE healthcare) according to manufacture’s instructions. After 48 hours treatment, the cells were immediately used for further experiments.

### Quantitative real-time RT-PCR

Total RNA was isolated from cell lines using RNeasy Mini Kit (QIAGEN GmbH, Hilden, Germany) according to the manufacture’s protocol. First strand cDNA was synthesized from 1.0 μg of total RNA using SuperScript® VILO cDNA synthesis Kit and Master Mix (Thermo Fisher Scientific Inc., Waltham, MA, USA). Real-time mRNA expression analysis of *KIAA1199* and a housekeeping gene (*GAPDH*) for control was performed using TaqMan^@^ Gene Expression Assays and Step One Plus real-time PCR system (Thermo Fisher Scientific Inc.) according to the manufacture’s instruction. The assay numbers for these genes were as follows: Hs01552124_m1 (*KIAA1199*); and Hs02758991_g1 (*GAPDH*).

The relative quantification was given by the Ct values, determining the reactions for target genes and an internal control gene in all samples.

### Cell migration assay

The migratory ability of cells was determined by transwell cell migration assay using cell culture inserts equipped with a filter membrane containing 8 μm pores (BD Biosciences, Franklin Lajes NJ). The lower chamber was filled with RPMI1640 containing 10% FBS. The upper chamber was filled with 2.0 × 10^4^ cells (for PANC-1) or 4.0 × 10^4^ cells (for BxPC-3) in the RPMI1640 containing 1% FBS. After 24 h incubation, the cells remaining on the upper side of the filters were removed. The cells on the bottom surface of the membrane were stained with hematoxyline and eosin and the number of cells that had migrated to the bottom surface of the membrane were counted in five randomly selected microscopic fields in each samples. Cell migration assays were also performed in the presence of an IL-1ß or NS-398 which was added to lower chambers at the beginning of assay.

### Cell proliferation assay

The capability of cell proliferation was measured by MTT assay. Cells were plated at 3,000 cells/well in 96-well plates and incubated. Then cells were incubated with 10 μl MTT (SERVA Electrophoresis, Heidelberg, Germany) for 4 h at 37°C and 200 μl DMSO (Wako, Osaka, Japan) was pipetted to solubilize the crystal product. The absorbance of each well was measured with a microplate reader at a wavelength of 570 nm.

### Transfection of KIAA1199

The KIAA1199 expressing plasmids were kindly gifted from Dr. Giancarlo Marra (University of Zurich) [27]. PANC-1 was transfected with the recombinant plasmid pcDNA3.2V5DEST-KIAA1199 vector (KIAA1199 expression vector) or pcDNA3.2V5DEST empty vector (control vector) using Lipofectamine 2000 (Thermo Fisher Scientific Inc.) according to manufacture’s instructions. The clones were selected and maintained in medium containing G418 (0.4 mg/ml) (Life Technologies).

### Western blot analysis

The cells were harvested and total protein was extracted with PRO-PREP protein extraction solution (iNtRON Biotechnology, Gyeonggi-do, South Korea), protein concentration was determined by the BCA (bicinchoninic acid) protein assay Kit (Thermo Fisher Scientific Inc., Waltham, MA, USA). Equal amount of protein were subjected to 4-15% Mini-PROTEAN Precast Gel (Bio-Rad, Philadelphia, PA, USA) and transferred on to PVDF membranes (ATTO, Tokyo, JAPAN). Membranes were blocked for 1h with 1% non-fat milk in TBST buffer at room temperature, then were incubated with antibodies against KIAA1199 (Proteintech Group, Rosemont, IL, USA) and ß-actin (Proteintech Group) for overnight at 4°C or 1h at room temperature, followed by incubation with secondary antibodies (Proteintech Group) for 1h at room temperature. The proteins were visualized using an ECL Western Blotting Detection System (GE healthcare, Buckinghamshire, England).

### Cell invasion assay

Cell culture inserts equipped with a filter membrane containing 8 μm pores (BD Biosciences) were coated with 20μg of Matrigel (Corning, NY, USA). The lower chamber was filled with RPMI1640 containing 10% FBS. The upper chamber was filled with 5.0 × 10^4^ cells in the RPMI1640 containing 0.1% FBS. After 24 h incubation, the cells remaining on the upper side of the filters were removed. The cells on the bottom surface of the membrane were stained with hematoxyline and eosin and the number of cells that had invaded to the bottom surface of the membrane were counted in five randomly selected microscopic fields in each samples.

### Measurements of LMW-HA concentrations

The cells (1.0 × 10^5^ cells/ml) were cultured in a serum-free medium (RPMI1640 without FBS) for 48 hours and the culture medium was collected for measurements of HA concentrations. The culture medium sample was centrifuged at 14000 g for 10 minutes through a Amicon Ultra-0.5 Centrifugal Filter Devices (MilliporeSigma, Darmstadt, Hessian, Germany) with a 100 kDa cutoff and the LMW-HA (<100 kDa) were collected [9, 28]. The concentration of LMW-HA was measured using Quantikine ELISA Hyaluronan Immunoassay (R&D Systems Inc., Minneapolis, MN, USA). Assays were triplicated and the average concentrations were determined.

### Statistical analysis

Statistical analyses were performed using SPSS statistical software version 21.0 (SPSS, Chicago, Illinois, USA). Student’s*t* test and Mann-Whiteney U test were used for group comparison. A P-value of < 0.05 was considered statistically significant.

## Abbreviations

PDAC: Pancreatic ductal adenocarcinoma
ECM: extracellular matrix
HA: Hyaluronan
LMW-HA: low-molecular-weight HA
HMW-HA: high-molecular-weight HA
HASs: HA-synthesizing enzymes
HYALs: hyaluronidases
IL-1ß: interleukin-1ß
COX-2: cyclooxygenase-2
FBS: fetal bovine serum.
